# A Functional Recovery Program for Femoroacetabular Impingement in Two Professional Tennis Players: Outcomes at Two-Year Follow-Up

**DOI:** 10.3390/jfmk10030309

**Published:** 2025-08-10

**Authors:** Francesca Campoli, Lucio Caprioli, Ida Cariati, Saeid Edriss, Cristian Romagnoli, Vincenzo Bonaiuto, Giuseppe Annino, Elvira Padua

**Affiliations:** 1 Department of Human Science and Promotion of Quality of Life, San Raffaele Rome University, 00166 Rome, Italy; cristian.romagnoli@uniroma5.it (C.R.); elvira.padua@uniroma5.it (E.P.); 2 Sports Engineering Laboratory, Department of Industrial Engineering, University of Rome Tor Vergata, 00133 Rome, Italy; lucio.caprioli@uniroma2.it (L.C.); saeid.edriss@alumni.uniroma2.eu (S.E.); vincenzo.bonaiuto@uniroma2.it (V.B.); 3 Department of Systems Medicine, “Tor Vergata” University of Rome, 00133 Rome, Italy; 4 Human Performance Laboratory, Centre of Space Bio-Medicine, Department of Medicine Systems, University of Rome Tor Vergata, 00133 Rome, Italy; giuseppe.annino@uniroma2.it

**Keywords:** hip injury, FAI, tennis, ATP players

## Abstract

**Background**: Femoroacetabular impingement (FAI) increasingly affects professional and amateur tennis players. Therefore, understanding the optimal approach to follow in specific cases plays an important role in the return to sports activity. **Methods**: This case report describes a two-stage functional recovery program implemented by two professional tennis players returning to practice. One player (a) underwent two arthroscopic surgeries for FAI and reconstruction of the damaged acetabulum labrum, while the other (b) took a stop period due to pain symptoms related to coxarthrosis and FAI. The subjects were monitored through pain perception questionnaires and mobility tests. An interview was conducted two years after the injury. **Results**: In both cases, at the end of the program, the subjects had no pain during tennis practice and gained more than 90% of range of motion in hip extension. However, both interrupted the program, experiencing painful symptoms again. At a two-year follow-up, the subjects were no longer involved in professional sports. The factors perceived by the two players were investigated and presented. **Conclusions**: The program proved beneficial, as both athletes returned to sports practice without pain symptoms and with hip mobility close to physiological. Nevertheless, the pain arose after the program ended and continued over time. Several aspects connected to the injury might have significantly affected early retirement from professional activity.

## 1. Introduction

The modern tennis game creates high loads on the hip joint [1]. For this reason, in recent years, an increasing number of hip injuries in tennis have been reported in the literature [2,3,4] and have been noted among elite tennis players. In addition, some tactical situations, such as defensive open stance, raised angles, and hip loading, potentially increase the risk of hip injuries [5]. This type of issue has been reported to occur in 8% to 27% of tennis players [6] with a substantial increase over the past decade [3]. A hip screening conducted on elite young tennis players found that 62% of the subjects in the sample had a hip “at risk” for hip impingement based on FABER test positivity or decreased hip internal rotation (>5 degrees) compared to the contralateral [7].

Several elite tennis players suffer or have suffered in their careers from hip problems, and it has been reported that one of the most common among male professional players is the femoroacetabular impingement (FAI) [8].

Femoroacetabular impingement is a condition of abnormal contact between the proximal femur head and the acetabulum. It is a cause of hip pain and degenerative change that can lead to acetabular labral tears and cartilage damage, and consequently has the probability of developing early osteoarthritis [9,10]. There are two types of impingements: CAM and Pincer [11]. CAM impingement results from abnormal morphology of the proximal femur, usually at the femoral head, and this type is more common in young male athletes. Conversely, pincer impingement is due to abnormal morphology or orientation of the acetabulum and is more common in middle-aged women [12]. FAI is a condition that can affect all individuals, but has a higher incidence among physically active people. It appears to be very much related to sports practice [13,14].

Some studies have indicated a probable dose-response relationship between the frequency of playing certain sports, such as soccer, ice hockey, and basketball, during growth and the increased risk of developing the femoral head-neck deformity associated with CAM-type FAI [15,16,17]. In fact, soccer players who practiced more than three times a week before age twelve showed almost three times the risk of having a CAM morphology in adulthood than those who practiced three or fewer days of physical activity before age twelve [18]. Other causes of impingement pain have been recognized, such as extra-articular hip impingement. These include ischiofemoral, anterior inferior iliac spine/subspine, iliopsoas, and greater trochanteric-pelvic impingement [7,19]. Arthroscopic surgery has become a procedure adopted by athletes of all ages to resolve FAI. Still, the functional recovery of athletes following arthroscopic surgery for FAI is often complex [20,21]. A careful, systematic approach is needed to ensure a better functional recovery and the player’s return to professional sports practice [22]. Various intervention approaches are reported in the literature, predominantly consisting of core and hip strengthening and physiotherapy [23,24,25]. Some studies have reported on the treatment of FAI in athletes and tennis players and the incidence of return to sports practice following hip arthroscopy surgery [26,27,28].

In support of the literature, this case report describes the adapted functional recovery program for two professional male tennis players returning to practice: one (a) after undergoing two arthroscopic surgeries for right hip FAI and reconstruction of the damaged acetabulum labrum, the other (b) after a stop period due to pain symptom for coxarthrosis and FAI in both hips, more pronounced to the left one. Discussions on the results achieved, return to sports practice, and a two-year follow-up are presented in this paper.

## 2. Materials and Methods

This case report was approved by the Internal Research Board of “Tor Vergata” University of Rome (Prot. No.: 003/23, 10 January 2023). All the procedures involved in this study were in accordance with the Declaration of Helsinki. The subjects gave their written consent to participate. They were in good health and were advised by physicians to return to sports practice before starting the program. The hip range of motion (ROM), flexibility of the back muscles, and pain perception were monitored daily during all six weeks of the program, as accurately described in the following sections.

### 2.1. Subjects

**Figure 1:**
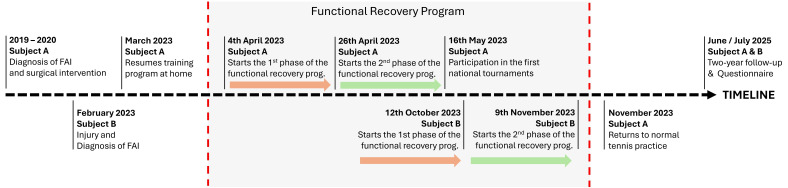
Summary timeline of the major clinical and sports events in the two subjects.

Two male athletes were recruited for this study. For simplicity and to ensure the anonymity of the subjects, hereafter referred to as “Subject A” and “Subject B”. The subjects were selected for convenience as they came in 2023 to a training center located in Velletri (Rome, Italy) with which the authors actively collaborate. Both subjects were ATP-ranked tennis players who had discontinued competitive activities following painful hip symptoms. The clinical diagnosis of FAI was made by medical specialists through MRI images. Both subjects performed the program described in this study in 2023, as shown in the timeline (Figure 1) in two distinct time-lags delimited in the figure by the vertical dashed lines.

About two years after the start of the program (26 months for Subject A, and 20 months for Subject B), an interview was conducted to investigate the professional activity status and the participant perspectives through the administration of a questionnaire described in the following paragraphs.

#### 2.1.1. Subject A

A 27-year-old male (body mass: 90.0 kg; height: 192 cm; BMI: 24.41) was involved in the study, identified as Subject A. Due to a CAM-type FAI in his right hip, the subject underwent two arthroscopic surgeries in 2019 at the age of 23, when he had an ATP ranking of #890 [29]. The aim of the intervention was to repair the damaged part of the labrum of the hip joint, reconstruct the removed fragment of the labrum on a collagen membrane, and build up the cartilage defect by autologous matrix-induced chondrogenesis (AMIC) technique. During the COVID-19 pandemic, he stopped training. In early 2023, he resumed sports practice and the training program at home in March 2023. He started the functional recovery program in Rome on the 4th of April 2023.

#### 2.1.2. Subject B

A 33-year-old male (body mass: 85.6 kg; height: 185.6 cm; BMI: 24.85) was involved in the study, identified as Subject B. Due to pain in his left hip, the individual stopped playing tournaments in February 2023 with an ATP ranking of #57 in doubles [29]. The subject, with coxarthrosis and CAM-type FAI in both hips, more pronounced to the left one, was directed to a conservative approach by physicians. After physiotherapist treatment, he began the functional recovery program on the 12th of October 2023, continuing the six prescribed monthly hyaluronic acid infiltrations.

### 2.2. Range of Motion

The modified Thomas test was used to assess hip extension and retraction of the Iliopsoas muscle [30,31]. An angle of −10.9 (±6) degrees was considered a physiological reference value for Iliopsoas flexibility in tennis players [32].

Images were acquired from a camera parallel to the measurement plane and aligned with the subject’s pelvis. The flexion angle was assessed using Kinovea (ver. 0.9.5, Boston, MA, USA) [33,34,35], measuring the angle between the thigh segment and the horizontal axis.

The flexibility of the posterior chain muscles was assessed through the Sit and Reach test [36] using a portable version of the instrument [37]. Descriptive statistics, pre-post percentage changes, effect sizes by Cohen’s d (ES) and 95% confidence intervals for ES (95% CI) were calculated by JASP Software (ver. 0.18.3, Amsterdam, The Netherlands) [38].

### 2.3. Pain Level

To classify the subject’s pain level, an eleven-point numeric rating scale (NRS), ranging from “0” (no pain) to “10” (the worst possible pain) [39,40]. Perceived pain levels during activities of daily living and during tennis practice were recorded.

### 2.4. Functional Recovery Program

The program was divided into two phases, each of which was three weeks, as explained in the following subsections.

#### 2.4.1. First Phase

The goal of this phase was to recover, control the pain, and continue to work on active and passive stretching of all muscle groups and strengthening core stability. During the first two weeks of the program, the subjects performed every day a mobility routine focused on lengthening the iliopsoas and increasing hip flexion-extension ROM by exercises shown in the Supplementary Materials (Figure S1), a strengthening program (Figures S2 and S3), and stretching (Figures S4 and S5) [41]. The strengthening exercises were aimed primarily at stimulating the extra rotator muscles of the pelvis, and in this, the use of an elastic band placed between the thighs at about one-third distal (Figures S2c,d and S3a,b) [41]. All exercises in this phase did not involve the use of weights. Two sets of ten repetitions of each strengthening exercise were performed, with twenty seconds of rest between them. After two weeks, the subjects proceeded with a week of unloading at home with a complete absence of tennis court practice, performing daily stretching exercises, three sets of twenty repetitions of abdominal, lumbar, and thirty-second front and side planks. It is to be reported that although on-court practice was not planned at this stage of the program, Subject A, on his own initiative, nevertheless tried to play on two occasions during the first two weeks, stopping after a few minutes at the onset of pain.

#### 2.4.2. Second Phase

This phase was focused on hip functionality recovery in parallel with returning to playing tennis and increasing workloads. The purpose was to control and reduce pain, mobilize, and recover the hip’s physiological joint flexibility, strengthen the hip’s stabilizing muscles, and simultaneously recover physical conditioning with gym and tennis practice. At this stage, some variations were made to the program, such as adding an elastic band between the thighs approximately at one-third distal in exercises Figure S3g,h to stimulate the extra rotatory muscles of the pelvis [41]. New exercises were added to the protocol shown in Supplementary Materials (Figure S6) [41]. The subjects repeated two sets of ten repetitions of each strengthening exercise. At the end of each training day, stretching exercises appropriate to the type of work done and two one-minute sets of Iliopsoas muscle stretching on the couch (modified Thomas, Figure S5d) were performed.

### 2.5. Two-Year Follow-Up Interview

Approximately two years after the injury, at exactly 26 months for Subject A and 20 months for Subject B, the professional activity status of the two individuals was verified directly on the ATP website [29]. A brief interview was conducted, and to understand their prospects, a questionnaire was administered in July 2025. In detail, the following points were investigated: (1) Perceived barriers to return to competition; (2) Motivation, (3) Pain experience over time; and (4) Subjective value of the program. The questionnaire (Appendix A) was administered to the subjects via a Google form (anonymous) with no special directions or instructions except the request to answer the questions truthfully. All questions in the questionnaire were optional, allowing respondents the freedom to choose whether to answer.

## 3. Results

### 3.1. Range of Motion

**Figure 2:**
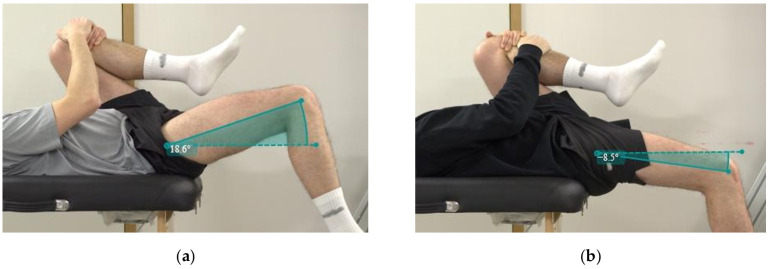
Modified Thomas test execution by Subject A: (**a**) on the first day of the program; (**b**) on the 14th day of the second phase of the program.

**Figure 3:**
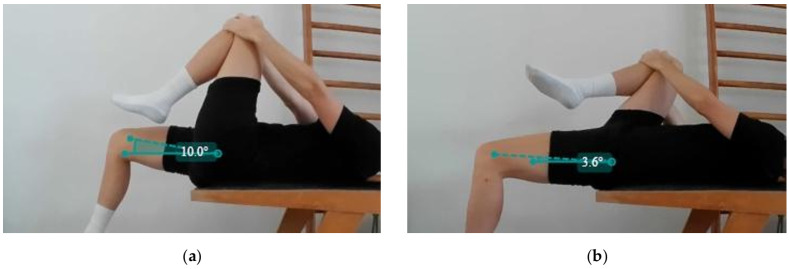
Modified Thomas test execution on the first day of the program by Subject B: (**a**) Right thigh extension; (**b**) Left thigh extension.

On the first day of the program, the modified Thomas test on Subject A indicated an angle of +18.6 degrees in the right hip, resulting in a deviation of 29.5 degrees from the average value of flexibility in tennis players (Figure 2a). Subject B showed an angle of +10.0 degrees in the right thigh (Figure 3a) and +3.6 in the left one (Figure 3b).

At the end of the program, an average improvement of 113.8% was found in the hip flexibility of the two subjects (ES: 5.7; 95% CI: 0.7–11.0) as reported in Table 1.

In detail, an increased ROM of the hip extension was achieved in both subjects, approaching the physiological range. In particular, Subject A gained more than 27 degrees of ROM in the extension of the right thigh (Figure 2b), and Subject B showed an increase in both sides (Right Thigh Δ: 19 degrees; Left Thigh Δ: 23 degrees). It should be pointed out that Subject B had reduced mobility in the left thigh flexion movement, evident in Figure 3a, and partially recovered at the end of the program.

**Figure 4:**
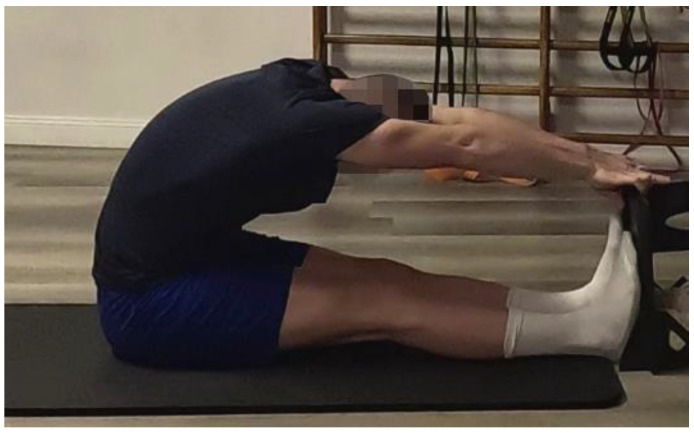
Sit and Reach Test performed at the end of the program by Subject B, using a portable version of the instrument developed by Panzarino and Padua [37].

Subject “A” had any significant retractions at the posterior chain muscles and showed good flexibility in the bending test, while Subject B had retractions at the lumbar muscles, and evident compensations were found. At the end of the last week, the Sit and Reach Test showed an increase in flexibility of 12.5 cm in Subject B (Figure 4).

### 3.2. Pain Level

At the end of the program, significant improvements were observed in both subjects. They could return to normal practice with pain levels controlled or absent, even as the workload increased. Indeed, during practice on all days of the 6th week, the subjects reported a pain level of 0/10, as shown in Table 2.

The pain perceived during daily activities was also reduced (Table 1). On the 7th day of the second phase (following the day with a higher training load), Subject A experienced adverse pain flare-ups rated 3/10 groin pain outside the court during the walking phase. However, this remains a remarkable improvement compared to the first weeks of training. The players also reported feeling better on the court.

### 3.3. Two-Year Follow-Up

Two years after the injury, the players continued their daily activities without experiencing any problems, but neither did they practice any competitive activities. They both reported that they stopped the mobility program and experienced discomfort again a few weeks after the second phase, and every time they tried to play tennis again in the following months. Investigating perceived barriers to return to competition, the responses provided by players are reported.

To the question, “What do you believe were the primary obstacles or challenges that prevented your return to competitive play within the two-year follow-up period?” they answer:

- “The influence of people from the close environment distracts from the competitive mode.”
- “The mix of different doctors (physiotherapist) opinions so was searching for lot of opinions and tried everything. Psychologic as well was not easy because to feel pain for such a long time was not easy for me and it is not easy now as well but trying not to think about it and doing everything AO i can be pain free and return on the court.”

**Figure 5:**
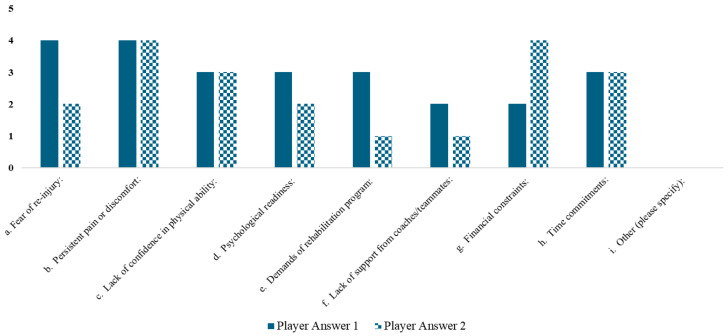
Answers to the question, “To what extent did the following factors particularly hinder your progress or discourage you from returning to competitive play?” were reported by the two players who answered the questionnaire anonymously. (on a scale of 1 to 5, where 1 = not at all, 5 = very much).

The answers to the question, “To what extent did the following factors particularly hinder your progress or discourage you from returning to competitive play?” are reported in Figure 5.

Investigating the motivation for returning to play, they both declared to be at the beginning of the functional recovery program, motivated or extremely motivated (4, 5/5; on a scale of 1 to 5, where 1 = not at all motivated, 5 = extremely motivated). The primary motivators for wanting to return to competitive play were passion and love of sport. In addition, they both liked the lifestyle of a professional athlete, and they had the confidence to achieve much greater competitive results.

To the question, “How did your motivation to return to competitive play change over the two-year follow-up period?”, one answered that age, the influence of people in his close environment, affected his motivation, while the other declared that the motivation was always high. Still, the presence of pain made him think less about tennis.

About the external factors, one declared that their wife was not supporting traveling for long periods, while the other always had the support of the family during the rehabilitation; he never had any issue. However, after 2.5 years, he does not play anymore.

About the pain experienced over time, they both declared that after the injury and during the initial phases of the functional recovery program it was very painful and this pain remains in the next two years, even in in a different way (“pain was like a sharp pinch in front of my hip flexor, and it was strong pain, so this type of pain was feeling for longer period, when I was during recover and i am still doing it i am feeling better in that part”).

To the question, “How did your pain experience impact your daily life, functional recovery program exercises, and overall outlook on returning to competitive play?” They answered as follows:

- “Not good. Motivation dropping each time the pain is getting bigger.”
- “The pain impacts my daily life a lot because was feeling it during my sleep and during walk or moving so couldn’t do normal things outside of the court. Program exercises were helping, but it is not easy mentally to do the same kind of things repeatedly for 2 Years and more. You must stay mentally strong, be motivated to keep the works so was not easy overall.”

To the question, “Were there any specific strategies or interventions that you found particularly helpful or unhelpful in managing your pain?”, they answered as follows:

- “Maybe lack of discipline with prevention exercise and recovery.”
- “Pain killers helped me little bit with pain, mobility exercise and relaxing tissue around the hip helped but not like that don’t feel it.”

In the last section, about the subjective value of the program, they evaluated the program as 4/5 on a scale of 1 to 5, where 1 = not at all valuable, 5 = extremely valuable.

About the aspects of the functional recovery program, they reported education, physical therapy, and mobility to be most beneficial.

To the question, “What aspects of the functional recovery program do you believe could be improved to better support athletes in returning to competitive play?” one of them answered, “I have no idea”, while the other, “I think working with physiotherapist that uses the hands are the most important things what physio can use”.

Finally, they both declared felt adequately supported by the program staff (“Yes, very much” and “Yes, every trainer or every physiotherapist tried to do their best”), and they advised other athlete going through a similar injury and functional recovery program “to stay motivated and show a lot of discipline” and “to stick with one person if you think he can help you send that you think is very good and that you have trust in him. In my opinion changing lot of opinions, doctors is not good way”.

## 4. Discussion

This report illustrates a two-stage functional recovery program carried out by two professional tennis players who had stopped playing sports due to painful symptoms from FAI and were advised to return to sports practice by physicians.

Although with different starting conditions, one returning from surgical intervention (Subject A) and the other from a conservative approach (Subject B), both had important muscle retractions and compensations in performing simple exercises such as squats and experienced pain while playing tennis. The main goal of the six-week program was the recovery of mobility, pain control, and gradual return to sports practice.

The athletes achieved satisfactory results, returning to tennis practice without pain and with joint mobility approaching the physiological range [32] in five weeks. Specifically, Subject A gained more than twenty-seven degrees of ROM in right hip extension, while Subject B showed almost twenty degrees of improvement in both lower limbs. Interestingly, Subject B had a limitation in left thigh flexion, which was partially recovered by the end of the program. In addition, the improvement in posterior chain flexibility in Subject B, as measured by the Sit and Reach test [36,37], suggests a positive impact of the program on the flexibility of back muscles as well. The protocol effectively eliminated pain during tennis and controlled it during daily activities, enabling a return to competitive tennis with a controlled or absent level of pain despite the increased workload. Subject A reported an episode of groin pain while walking only following a day of high training load. Both players reported feeling better on the court.

However, despite the positive outcomes, both subjects no longer practiced professional activity two years after the injury. Indeed, the interventions carried out to increase the range of motion did not prove to be effective in permanently eliminating the pain, and once they are discontinued, the pain recurs.

The main factors that they believed limited their return to competition were, above all, the influence of people close to them who distracted them from the competitive mode and the mixture of different opinions from other doctors.

Among others, the following factors were noted: persistent pain or discomfort; fear of re-injury; lack of confidence in physical ability; financial constraints (for one of them); and time commitments.

Both stated that they were highly motivated at the start of the program; however, persistent pain symptoms, which recurred at the end of the program and continued over the following months, significantly impacted their motivation and consistency in following the training programs.

The players rated the program as valuable and felt very supported by the staff. If they were to advise another player in the same situation, they would recommend staying motivated and continuing with discipline the program they feel is best suited to them, following a single guide and not constantly changing opinions and doctors.

This case report provides insight into the complexity of the functional recovery process for FAI and the return to tennis practice after hip surgery or conservative treatment. Although most professional athletes return to sports after hip surgery [28,42,43], this approach is not simple; complete recovery is not always achieved [44], and several factors can influence the return to competition. Although the literature seems to favor a surgical approach over a conservative one, neither approach generally leads to complete functional recovery [45]. This study proposes a protocol for the management of FAI symptoms in reducing pain and improving hip function that is applicable in non-surgical management. However, future studies are needed to identify new training guidelines and biomechanically advantageous and less stressful playing techniques [46,47] to attempt to reduce the incidence of this issue.

### Future Implications and Limitations

This case report suggests that a well-structured functional recovery program can significantly improve ROM and reduce pain in tennis players with hip issues, facilitating a return to sports practice. However, both players did not maintain the discipline to continue the program over time, either due to the influence of outside people or because they tried to listen to other opinions and doctors. The outcome measures used (ROM, sit-and-reach, pain NRS) together with the return to normal practice were relevant; however, no other functional performance tests (CMJ, Spider Test, and on-court movement drills) were carried out.

The withdrawal from competitive activity at two years highlights the complexity of complete recovery for athletes at this level and raises several thoughts. Even the long process of meticulous recovery could be a sacrifice for a player already near the end of their career who may not continue to pursue competitive play.

Although this case report adds content to what is already in the literature, the presence of only two subjects limits the generalizability of the results. The follow-up indicates withdrawal from competitive activity and only presents the athlete’s perceived reasons and some psychological factors such as fear of recidivism, motivation, or other personal decisions, without an in-depth analysis. In addition, a detailed study aimed at reducing the risk of occurrence of these kinds of injuries with proper off-court training habits or how choosing less wearing and equally effective playing techniques can help safeguard players’ health [46].

Future research with larger numbers of participants is needed to evaluate the effectiveness of specific functional recovery protocols for tennis players with FAI and other hip disorders. In addition, inflammatory processes could be monitored throughout the entire process (e.g., laboratory markers and magnetic resonance imaging), thus providing objective evidence of the effectiveness of the conservative approach. Factors influencing return to long-term competitive play should be investigated in future studies, closely monitoring the various stages of returning to sports with short-term, middle-term, and long-term follow-ups [42], and the psychological aspects of the injury should also be better considered as well.

## 5. Conclusions

The proposed two-phase recovery program focused on pain control, recovery of mobility, and progressive increase in workload appears to have provided an effective approach for managing pain symptoms in these high-level athletes. The emphasis on strengthening core stability and extra-rotator muscles of the hip, combined with targeted stretching exercises, appears to align with the literature’s recommendations. However, the two-year follow-up reveals an important aspect: although they resumed normal activities, neither player was engaging in competitive activities. This finding raises several thoughts, and future research with larger numbers of participants is needed to investigate the psychological aspects of the injury.

## Figures and Tables

**Table 1:** Modified Thomas pre- and post-test results and improvements in ROM compared to the physiological value for tennis players reported in the literature [32].

	Modified Thomas Test		Δ from the Physiological Value		Percentage Changes
Conditions	Pre (°)	Post (°)	Pre (°)	Post (°)	
Subject A—Right	18.6	−8.5	29.5	2.4	91.9%
Subject B—Right	10.0	−9.0	20.9	1.9	90.9%
Subject B—Left	3.6	−19.4	14.5	−8.5	158.6%
Average	10.7	−12.3	21.6	−1.4	113.8%

**Table 2:** Pain subjects’ perception, on a numeric rating scale (NRS) from 0 to 10, during daily activities and tennis practice. NR—Not reported because the subject did not play.

**Pain NRS—Subject “A”**																						
**Phases**	**1st**	**2nd**																				
**Daily** **Levels**	**Median**	**1**	**2**	**3**	**4**	**5**	**6**	**7**	**8**	**9**	**10**	**11**	**12**	**13**	**14**	**15**	**16**	**17**	**18**	**19**	**20**	**21**
Daily activities	3	0	0	0	0	0	0	3	2	2	1.5	1.5	2	2	2	1	1	0	1	1	1	1
Playing Tennis	5	0	0	0	NR	0	0	0	0	0	NR	NR	0	0	0	0	0	NR	0	0	0	0
Playing Tennis	Yes	Yes	Yes	Yes	No	Yes	Yes	Yes	Yes	Yes	No	No	Yes	Yes	Yes	Yes	Yes	No	Yes	Yes	Yes	Yes
**Pain NRS—Subject “B”**																						
**Phases**	**1st**	**2nd**																				
**Daily** **Levels**	**Median**	**1**	**2**	**3**	**4**	**5**	**6**	**7**	**8**	**9**	**10**	**11**	**12**	**13**	**14**	**15**	**16**	**17**	**18**	**19**	**20**	**21**
Daily activities	2	1	2	1	1	1	1	1	1	1	1	1	1	1	1	0	0	0	0	0	0	0
Playing Tennis	NR	NR	NR	1	1	1	NR	1	1	1	1	1	1	NR	1	0	0	0	0	0	NR	0
Playing Tennis	No	No	No	Yes	Yes	Yes	No	Yes	Yes	Yes	Yes	Yes	Yes	No	Yes	Yes	Yes	Yes	Yes	Yes	No	Yes

## Data Availability

The data supporting the conclusions of this article are included within the article. The raw data and photos are not available for privacy and confidentiality reasons.

## Supplementary Materials

The following supporting information can be downloaded at: https://www.mdpi.com/article/10.3390/jfmk10030309/s1, Figure S1: Mobility exercise routine; Figure S2: Protocol of strengthening exercises; Figure S3: Protocol of strengthening exercises; Figure S4: Protocol of strengthening exercises; Figure S5: Stretching exercises; Figure S6: Strengthening exercises second phase.

## Abbreviations

The following abbreviations are used in this manuscript:

FAI	Femoroacetabular Impingement
ATP	Association of Tennis Professionals
NRS	Numeric Rating Scale
ROM	Range of Motion

## Appendix A

Questionnaire on Athlete Perspectives on Return to Play

Instructions: Please answer all questions thoroughly. Your honest and detailed responses are invaluable.

Section 1—Perceived Barriers to Return to Competition
  1. What do you believe were the primary obstacles or challenges that prevented your return to competitive play within the two-year follow-up period?
    (Please consider physical, psychological, social, and logistical factors)
  2. To what extent did the following factors particularly hinder your progress or discourage you from returning to competitive play?
    (Please rate on a scale of 1 to 5, where 1 = Not at all, 5 = Very much)
    - Fear of re-injury:
    - Persistent pain or discomfort:
    - Lack of confidence in physical ability:
    - Psychological readiness (e.g., anxiety, frustration):
    - Demands of the program:
    - Lack of support from coaches/teammates:
    - Financial constraints:
    - Time commitments:
    - Other (please specify):
Section 2—Motivation for Return to Play
  1. At the beginning of your functional recovery program, how motivated were you to return to competitive play?
    (Please rate on a scale of 1 to 5, where 1 = Not at all motivated, 5 = Extremely motivated)
  2. What were your primary motivators for wanting to return to competitive play?
    (e.g., love of sport, team camaraderie, personal achievement, professional goals)
  3. How did your motivation to return to competitive play change over the two-year follow-up period? Please describe any shifts, increases, or decreases in your motivation and what factors contributed to these changes.
  4. Were there any external factors
    (e.g., support from family/friends, professional expectations, perceived pressure) that influenced your motivation, either positively or negatively?
Section 3—Pain Experience over Time
  1. Please describe your pain experience immediately after the injury and during the initial phases of your functional recovery program.
  2. How did your pain level change throughout the two-year follow-up period?
    (e.g., did it decrease steadily, fluctuate, or remain constant?)
  3. How did your pain experience impact on your daily life, functional recovery program exercises, and overall outlook on returning to competitive play?
  4. Were there any specific strategies or interventions that you found particularly helpful or unhelpful in managing your pain?
Section 4—Subjective Value of the Program
  1. Overall, how valuable did you find the functional recovery program you participated in?
    (Please rate on a scale of 1 to 5, where 1 = Not at all valuable, 5 = Extremely valuable)
  2. What aspects of the functional recovery program did you find most beneficial?
    (e.g., physical therapy, psychological support, education, access to facilities)
  3. What aspects of the functional recovery program do you believe could be improved to better support athletes in returning to competitive play?
  4. Did you feel adequately supported by the program staff (e.g., trainers, physical therapists, doctors, psychologists)? Please describe.
  5. If you were to advise another athlete going through a similar injury and functional recovery program, what would be the most important piece of advice you would offer regarding their involvement in such a program?
