# Supplementary material for: A Functional Recovery Program for Femoroacetabular Impingement in Two Professional Tennis Players: Outcomes at Two-Year Follow-Up
Source: J Funct Morphol Kinesiol. 2025 Aug 10;10(3):309. doi: 10.3390/jfmk10030309 (PMC12371922; doi:10.3390/jfmk10030309)
Supplement: Supplementary file 1 [file jfmk-10-00309-s001.zip › jfmk-3749145-supplementary.pdf]

Supplementary material: Functional Recovery Program for FAI.

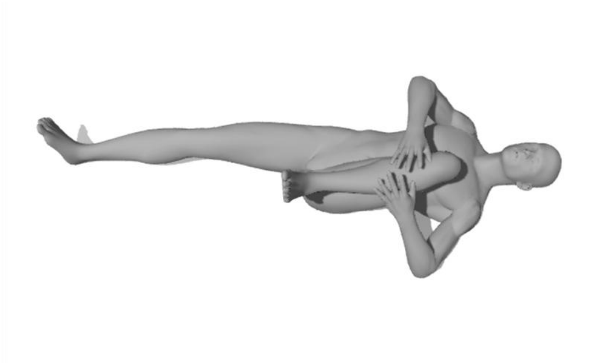

(a)

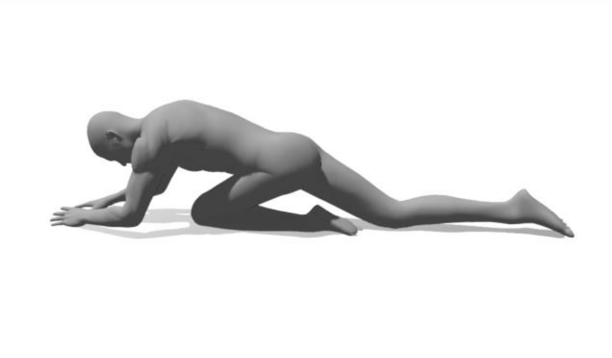

(b)

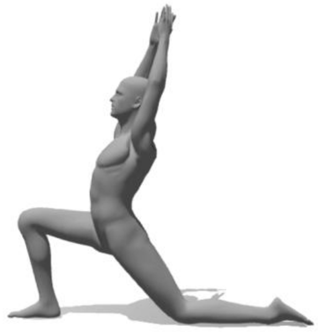

(c)

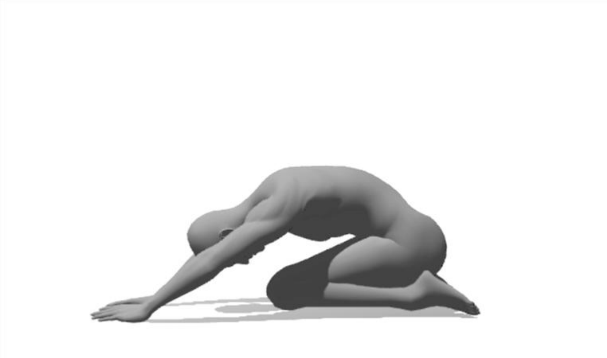

(d)

Figure S1. Mobility exercise routine: (**a**) Iliopsoas and gluteus; (**b**) gluteus; (**c**) Iliopsoas muscle; (**d**) back muscles.

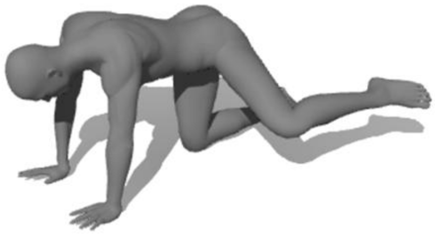

(a)

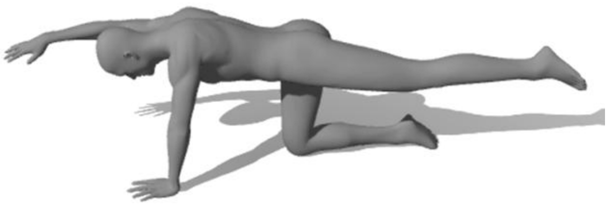

(b)

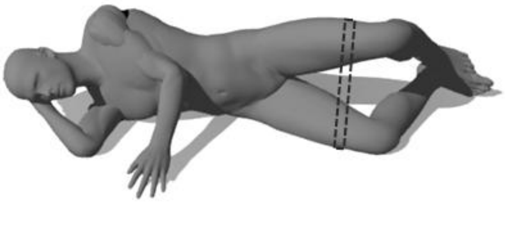

(c)

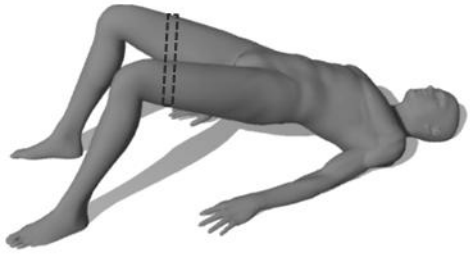

(d)

Figure S2. Protocol of strengthening exercises: (**a**) hip lift; (**b**) lumbar; (**c**) external hip rotation with elastic band; (**d**) bridge with elastic band.

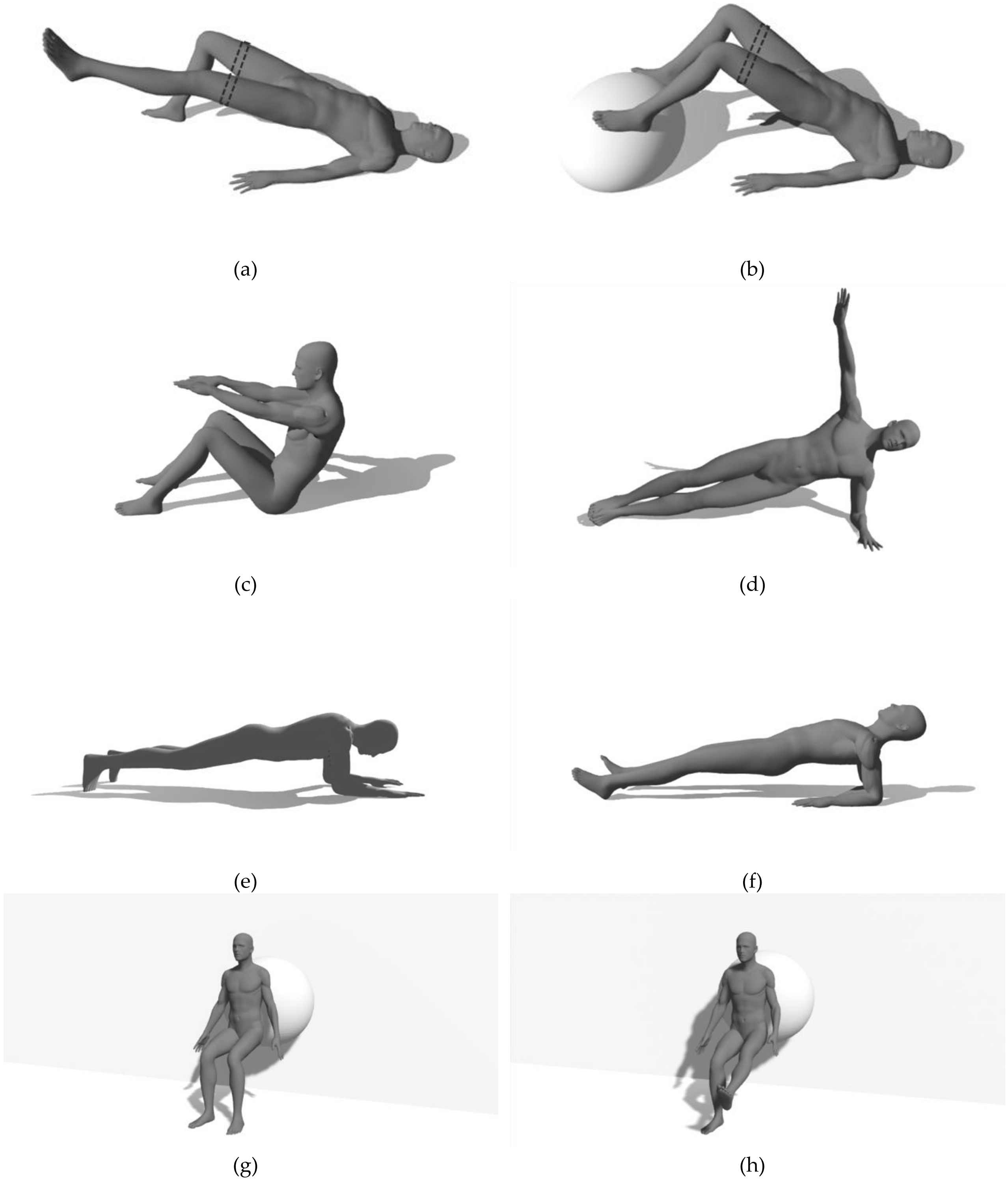

Figure S3. Protocol of strengthening exercises: (**a**) bridge exercise with knee extension; (**b**) bridge on the Swiss ball; (**c**) abdominals; (**d**) side plank; (**e**) plank exercise; (**f**) reverse plank; (**g**) wall sits with the Swiss ball; (**h**) wall sits with the Swiss ball and knee extension.

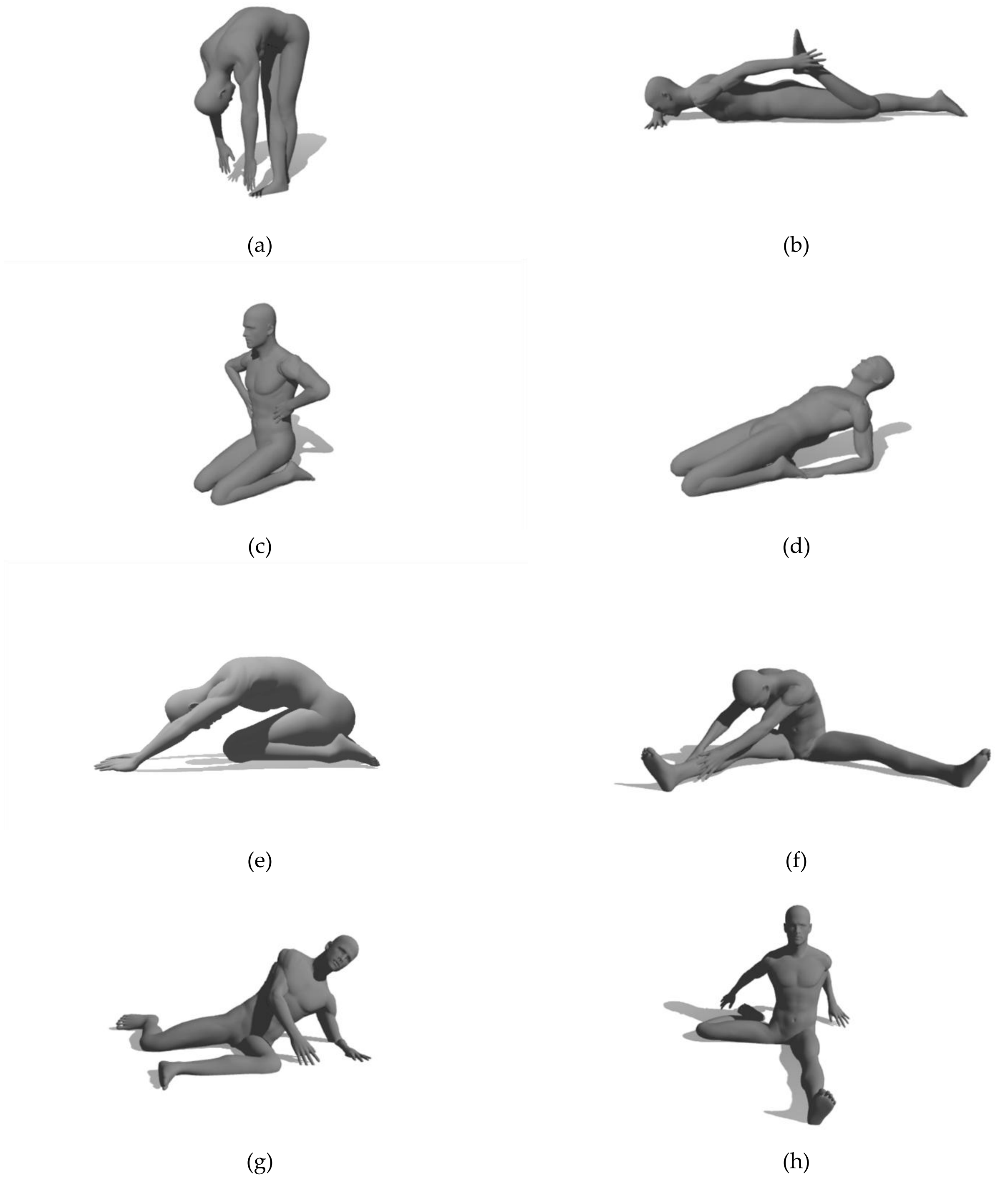

Figure S4. Stretching exercises: (**a**) gluteus, piriform and hamstring muscles; (**b**) quadriceps; (**c**) tibial; (**d**) tibial and quadriceps; (**e**) back muscles; (**f**) lumbar and hamstring muscles; (**g**) sartorius; (**h**) quadriceps, adductors and hamstrings muscles.

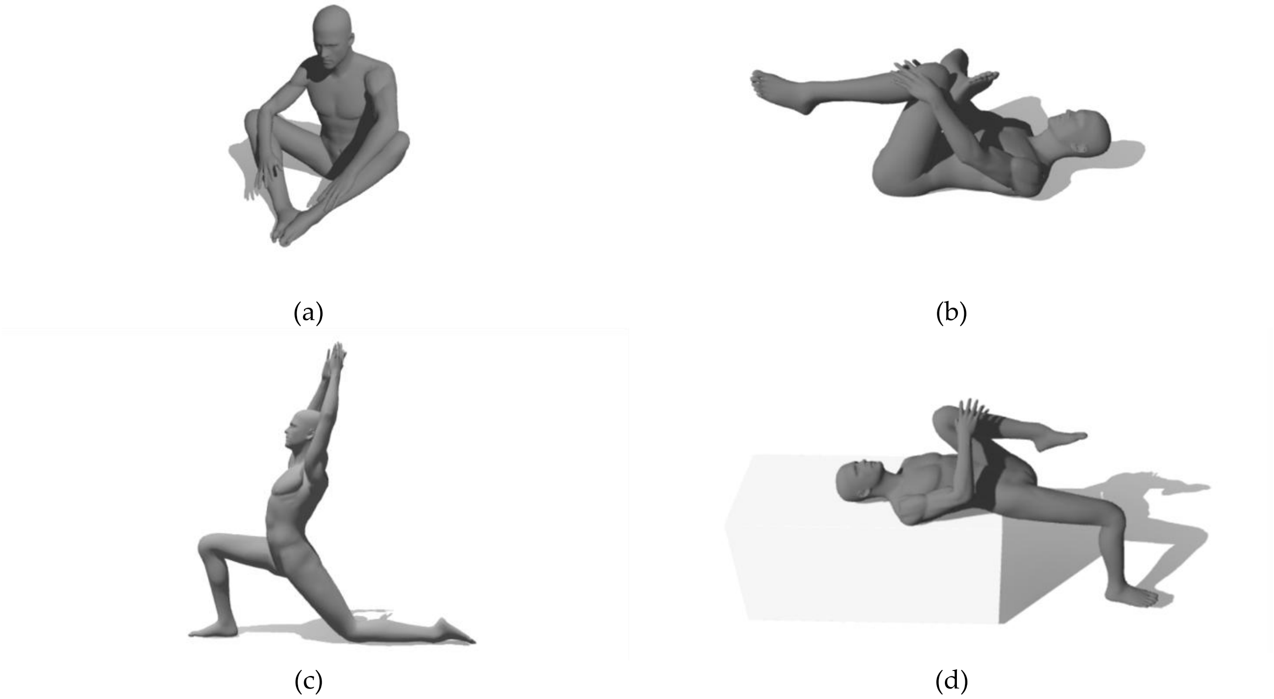

Figure S5. Stretching exercises: (**a**) Adductors muscles; (**b**) gluteus; (**c**) and (**d**) Iliopsoas muscle.

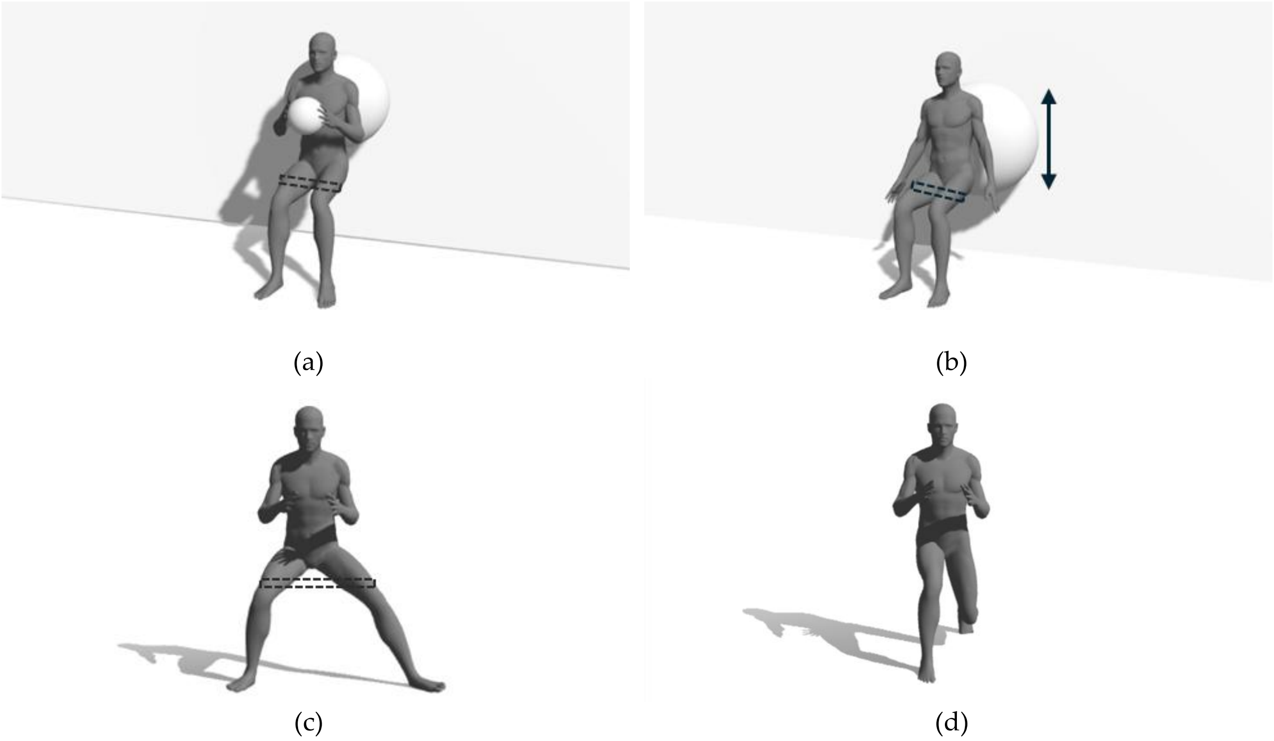

Figure S6. Strengthening exercises Second Phase: (**a**) wall sits with the Swiss ball and overload ; (**b**) half wall squat with the Swiss ball and elastic band; (**c**) side and diagonal walking with resistance band (**d**) walking lunge.
